# Author Correction: Marrying chemistry with biology by combining on-chip solution-based combinatorial synthesis and cellular screening

**DOI:** 10.1038/s41467-019-13725-x

**Published:** 2019-12-04

**Authors:** Maximilian Benz, Mijanur R. Molla, Alexander Böser, Alisa Rosenfeld, Pavel A. Levkin

**Affiliations:** 1 Karlsruhe Institute of Technology (KIT), Institute of Toxicology and Genetics (ITG), Hermann-von-Helmholtz-Platz 1, 76344 Eggenstein-Leopoldshafen, Germany; 2 Department of Chemistry, University of Calcutta, 92, APC Road, Kolkata, 700009 India; 3 Karlsruhe Institute of Technology (KIT), Institute of Organic Chemistry (IOC), Kaiserstraße 12, 76131 Karlsruhe, Germany

**Keywords:** Lipids, High-throughput screening, Combinatorial libraries

Correction to: *Nature Communications* 10.1038/s41467-019-10685-0, published online 28 June 2019.

The original version of this Article omitted ‘The Ministry of Science, Research and the Arts Baden-Württemberg (MWK), Grant FKZ 7533-7-11-10-07 (IWWB-28)’ from the acknowledgements and has since been adjusted to read:

The research was supported by the ERC Starting Grant (DropCellArray, 337077), ERC Proof-of-Concept Grant (CellScreenChip, 680913) and the Ministry of Science, Research and the Arts Baden-Württemberg (MWK), Grant FKZ 7533-7-11-10-07 (IWWB-28). We would like to thank Dr. Gerald Brenner-Weiß from IFG (KIT, Institute of Functional Interfaces) for providing training and access to MALDI-TOF mass spectrometry, and Dr. Nicole Jung from IOC (KIT; Institute of Organic Chemistry) for providing training and access to LC-MS.

This has now been corrected in both the PDF and HTML versions of the Article.

