# Supplementary material for: Marrying chemistry with biology by combining on-chip solution-based combinatorial synthesis and cellular screening
Source: Nat Commun. 2019 Jun 28;10:2879. doi: 10.1038/s41467-019-10685-0 (PMC6599004; doi:10.1038/s41467-019-10685-0)
Supplement: Supplementary file 1 — Supplementary Information [file 41467_2019_10685_MOESM1_ESM.pdf]

Supplementary Information for

Marrying Chemistry with Biology by Combining On-Chip Solution-Based Combinatorial Synthesis and Cellular Screening

Benz et al.

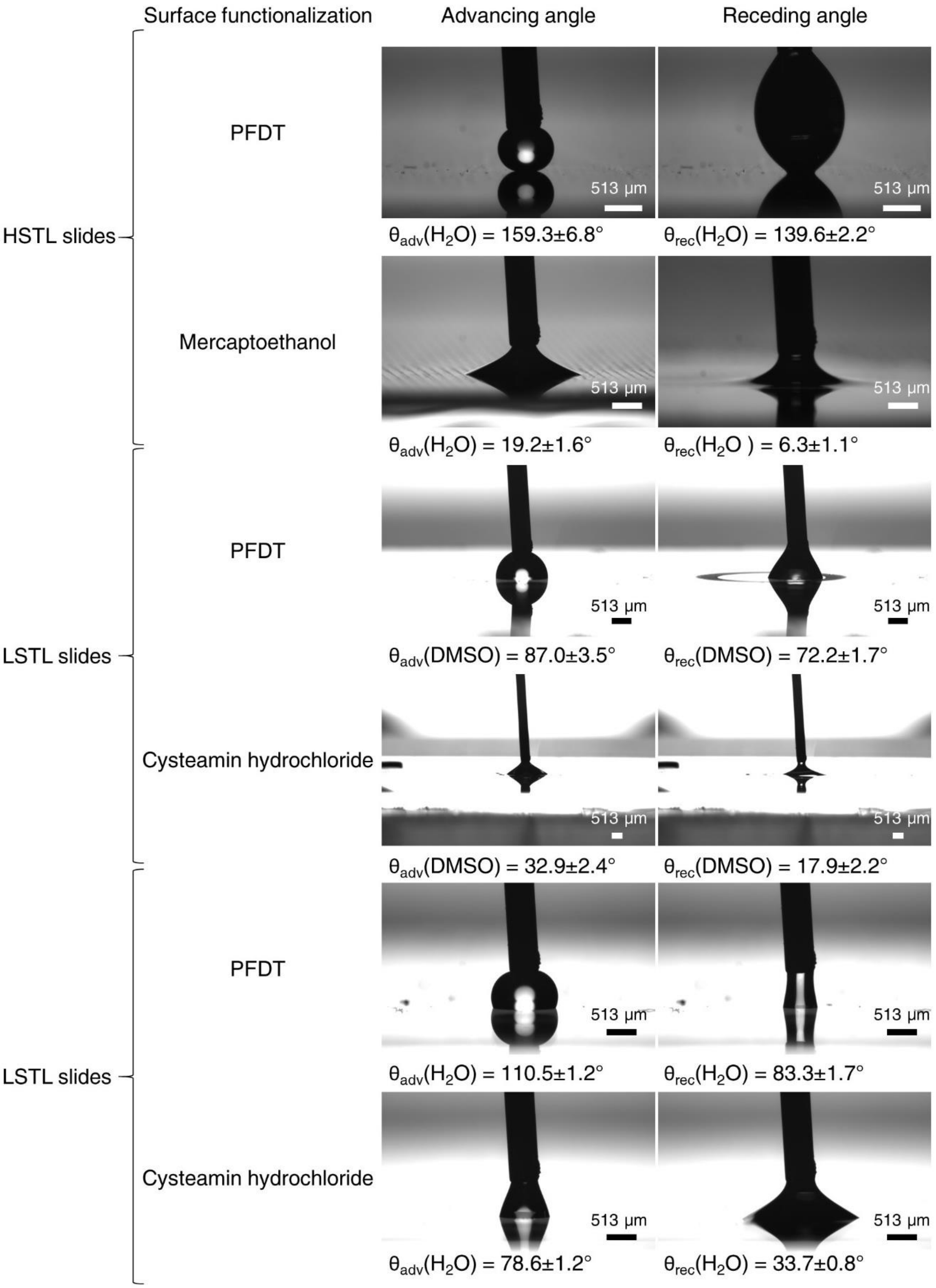

Supplementary Figure 1 | Water contact angle measurements. We observed advancing and redecing contact angle of several solvents (H_2O and DMSO) on different functionalized surfaces (perfluoro decanethiol (PFDT), cysteamine hydrochloride on rough polymer surfaces (HSTL slides) and bare glass surfaces (LSTL slides)). +/- values are standard deviations, n = 3 (number of replicates). Source data are provided as a Source Data file.

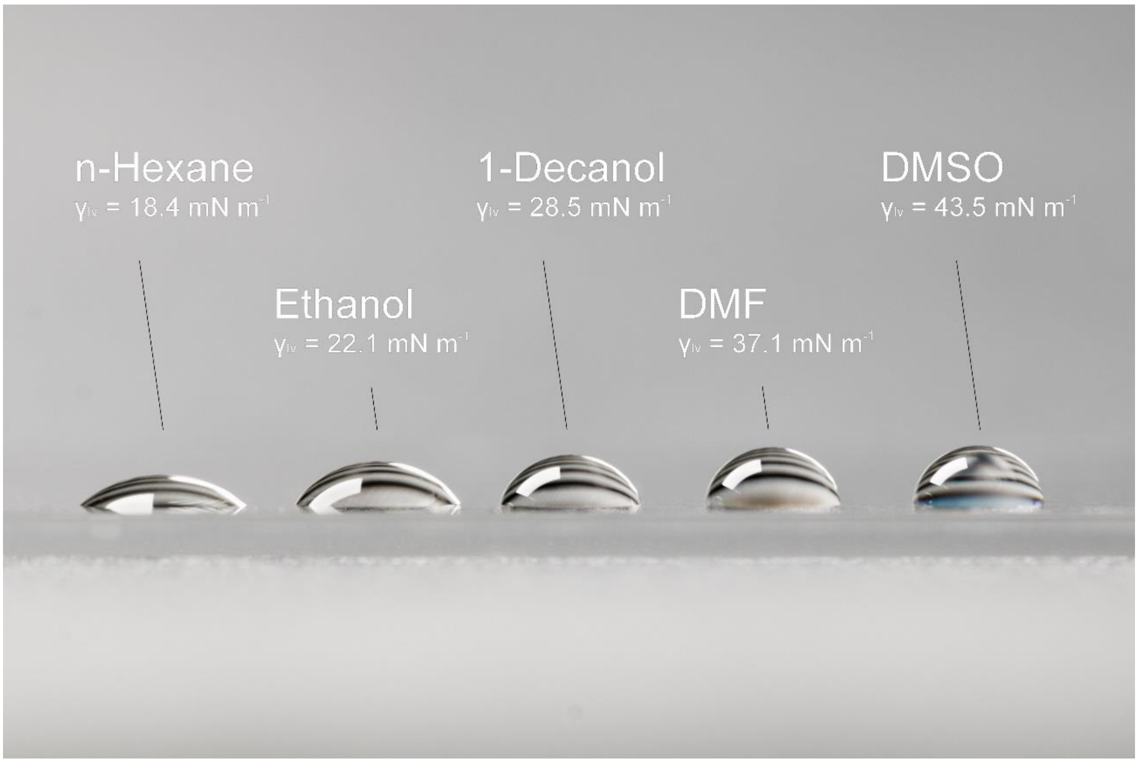

Supplementary Figure 2 | Photograph of droplets of various organic solvents on a Low Surface Tension Liquids (LSTL) slide. From left to right: n-hexane (γ_{lv} = 18.4 mN m^{-1}), ethanol (γ_{lv} = 22.1 mN m^{-1}), 1-decanol (γ_{lv} = 28.5 mN m^{-1}), DMF (γ_{lv} = 37.1 mN m^{-1}) and DMSO (γ_{lv} = 43.5 mN m^{-1}). Spot size: 2.83 mm; borders width: 1.67 mm; droplet volume: 5 µL.

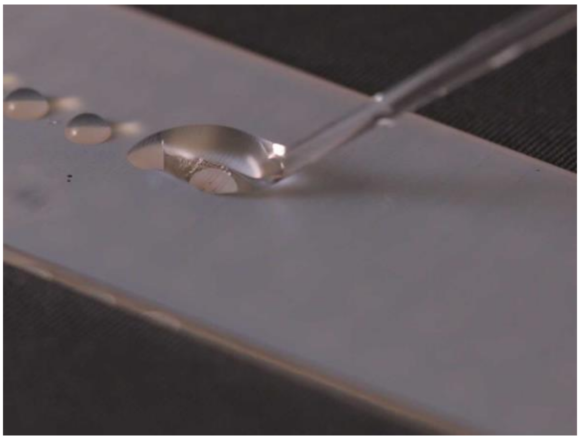

Supplementary Figure 3 | Rolling droplet. Solutions can be applied row-by-row by rolling a large droplet over the photochemically patterned surface. Huge differences in wettability between hydrophilic/omniphilic spots and surrounding hydrophobic/omniphobic borders lead to the formation of microdroplets.

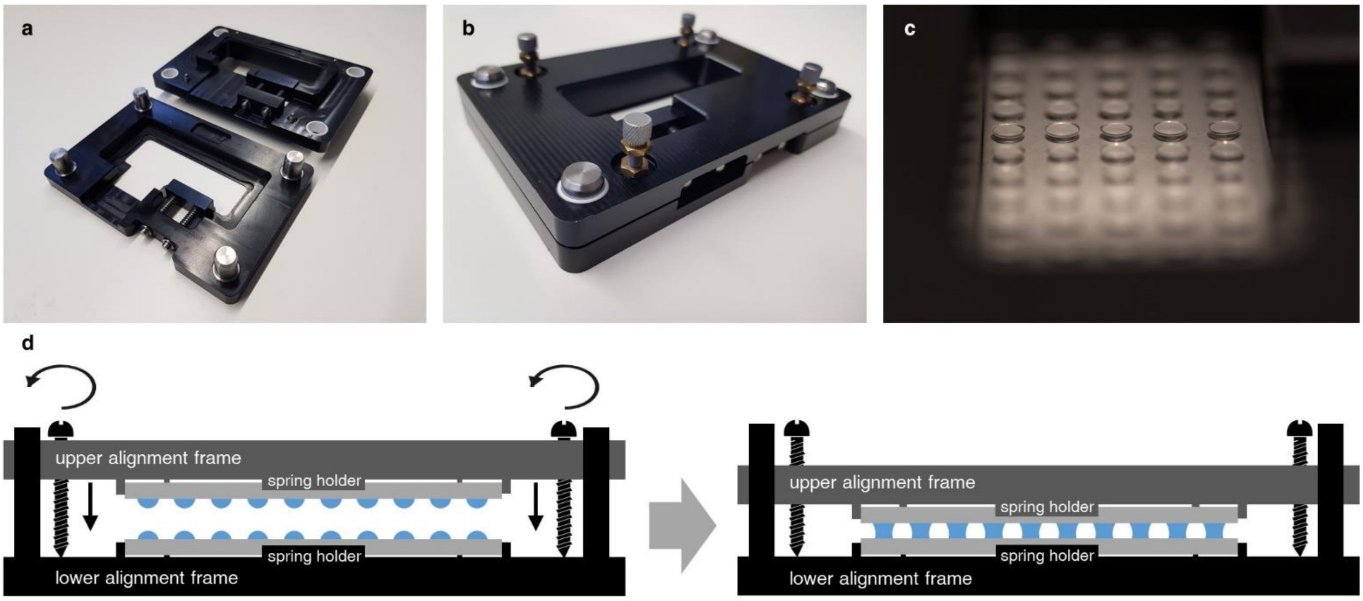

Supplementary Figure 4 | Alignment (sandwiching) device. (**a**) Photograph of the opened alignment device. (**b**) Photograph of the closed alignment device during the synthesis step. (**c**) Photograph of two sandwiched slides in the alignment device during the synthesis step. (**d**) Schematic describing the process of precise sandwiching of two droplet arrays. The alignment device consists of a lower and upper frame. Slide A is set into the lower frame of the alignment device and fixed with a spring holder. Slide B is set into the upper frame of the alignment device and fixed, too. The lower frame shows round rods in each edge of the frame, whereas the upper frame shows round recesses on the same positions. The upper frame is put on the lower frame so that the rods from the lower frame slide into the recesses of the upper frame and therefore fix both frames in the lateral position. The distance in height of both frames can be precisely controlled by four screws until the droplets of slide B merge with the droplets of slide A.

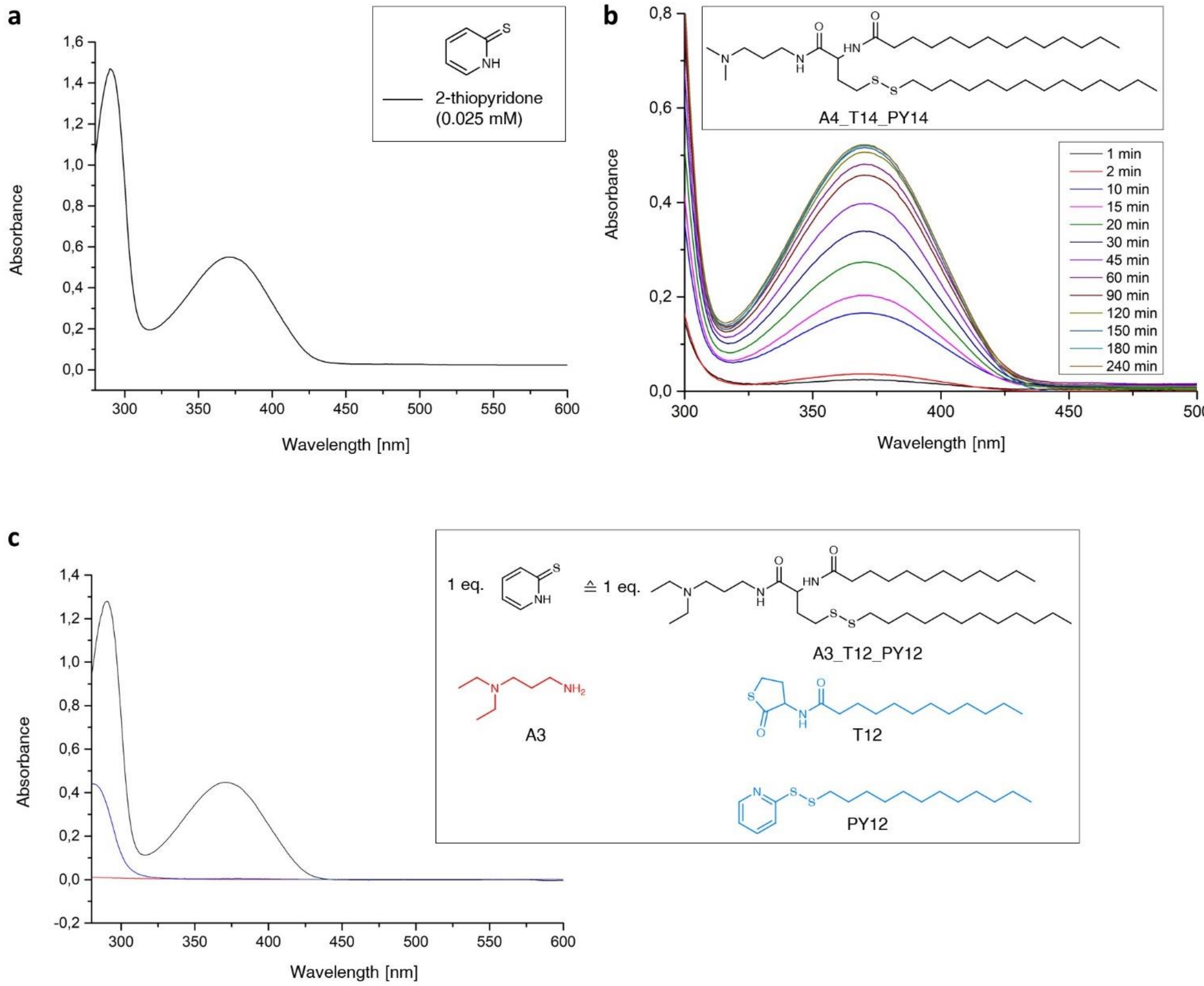

Supplementary Figure 5 | UV-Vis spectroscopy. (**a**) Spectrum of 0.025 mM 2-thiopyridone in DMSO/acetonitrile (1:10). Extinction coefficient was estimated to be 2040 M^{-1} cm^{-1} at 370 nm. (**b**) UV-Vis reaction kinetic measurements. UV-Vis absorbance of reaction mixture of sample A4_T14_PY14 was measured in range of 1-240 min. After approximately 2 h the reaction was completed.
(**c**) Exemplary UV-Vis absorbance spectrum of raw product A3_12_12 (black) compared to UV-Vis absorbance spectra of educts (A3, red / T12_PY12, blue). Optical depth: 1 cm. Source data are provided as a Source Data file.

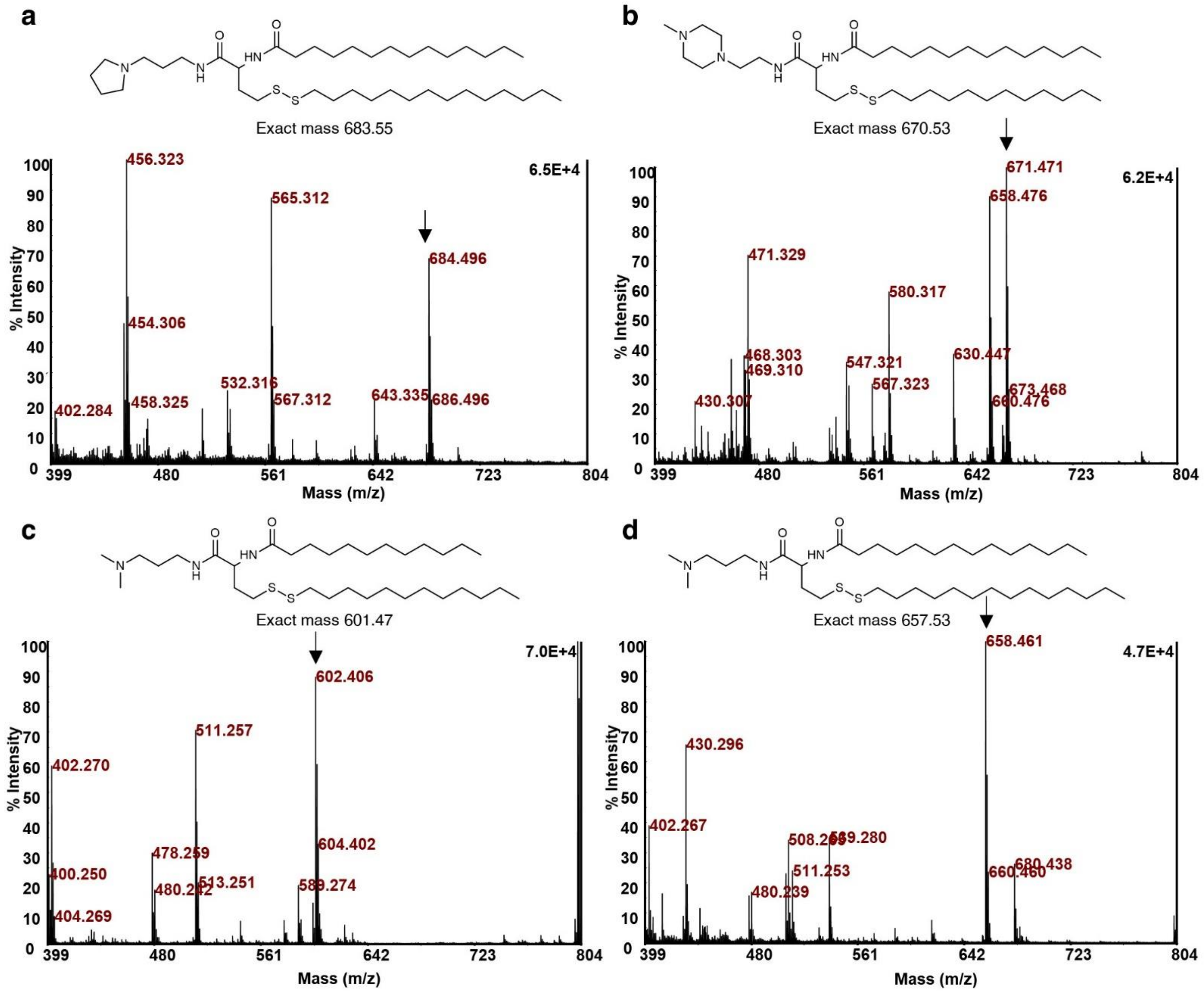

Supplementary Figure 6 | Exemplary mass spectra of several raw products. (**a**) A2_T14_PY14 – calculated mass (m/z): 683.55; found mass (m/z): 684.496. (**b**) A3_T14_PY12 – calculated mass (m/z): 670.53; found mass (m/z): 671.471. (**c**) A4_T12_PY12 – calculated mass (m/z): 601.47; found mass (m/z): 602.406. (**d**) A4_T14_PY14 – calculated mass (m/z): 657.53; found mass (m/z): 658.461. Source data are provided as a Source Data file.

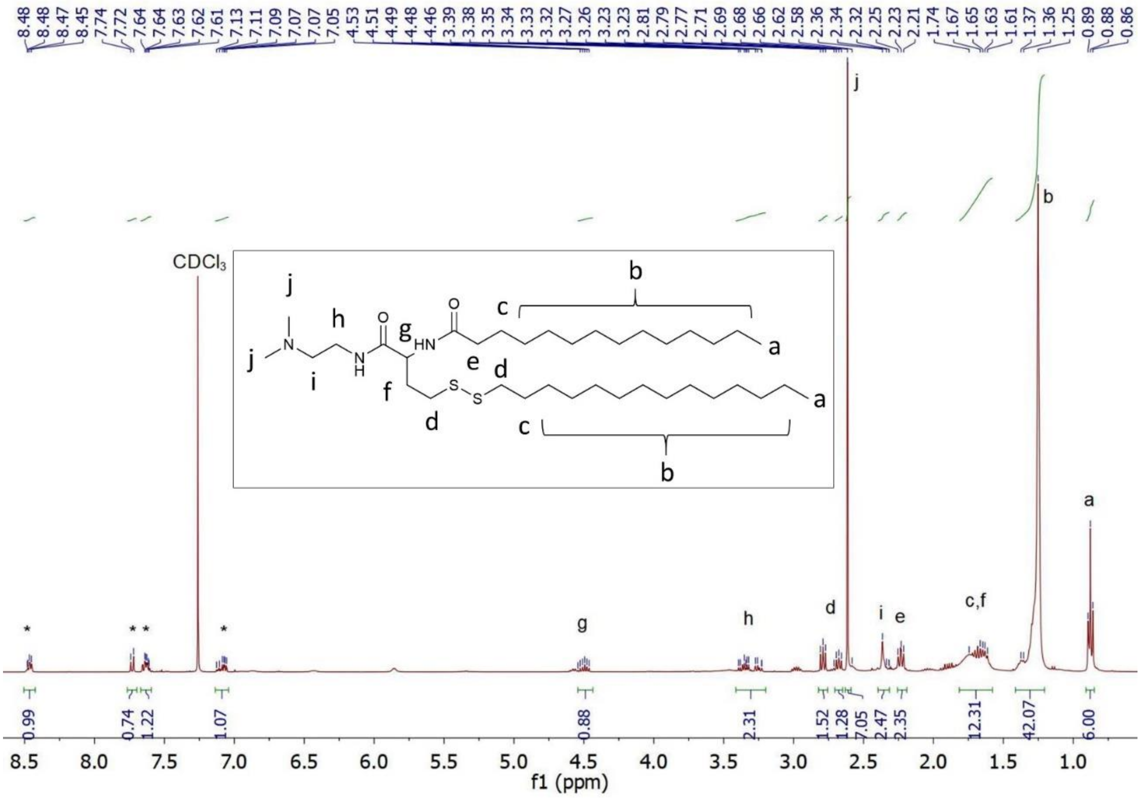

Supplementary Figure 7 | Exemplary ^{1}H NMR spectrum of on-chip synthesized raw product A5_T14_PY14. ^{1}H NMR (400 MHz, chloroform-*d*): δ 4.54-4.46 ppm (m, 1H), 3.39-3.23 (m, 2H), 2.81-2.77 (m, 2H), 2.71-2.66 (m, 1H), 2.62-2.58 (m, 7H), 2.81-2.58 (m, 2H), 2.36-2.32 (m, 2H), 2.25-2.21 (m, 2H), 1.74-1.61 (m, 12H), 1.37-1.25 (m, 42H), 0.89-0.86 (m, 6H). Peaks of by-product 2-thiopyridone labelled by *. Source data are provided as a Source Data file.

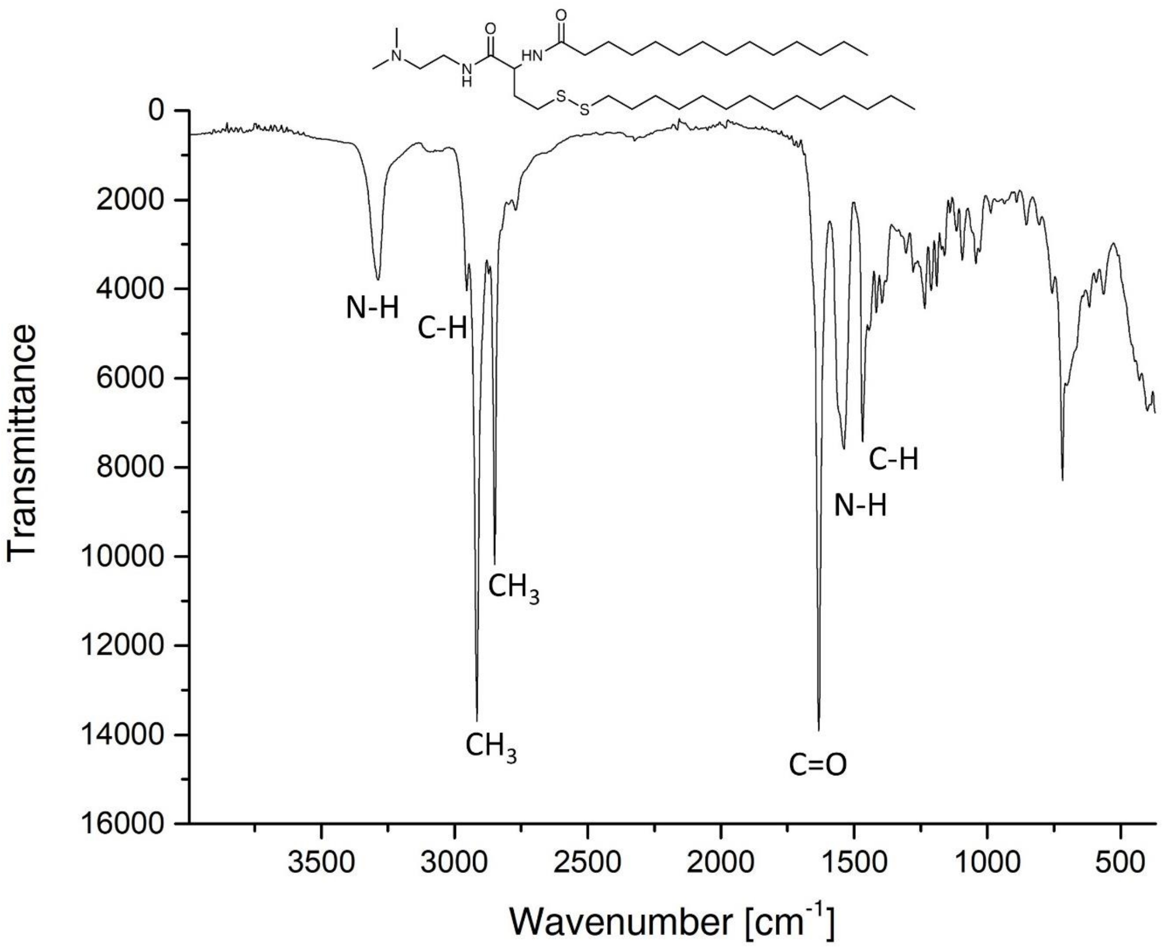

Supplementary Figure 8 | Exemplary IR spectrum of the product A5_T14_PY14. Source data are provided as a Source Data file.

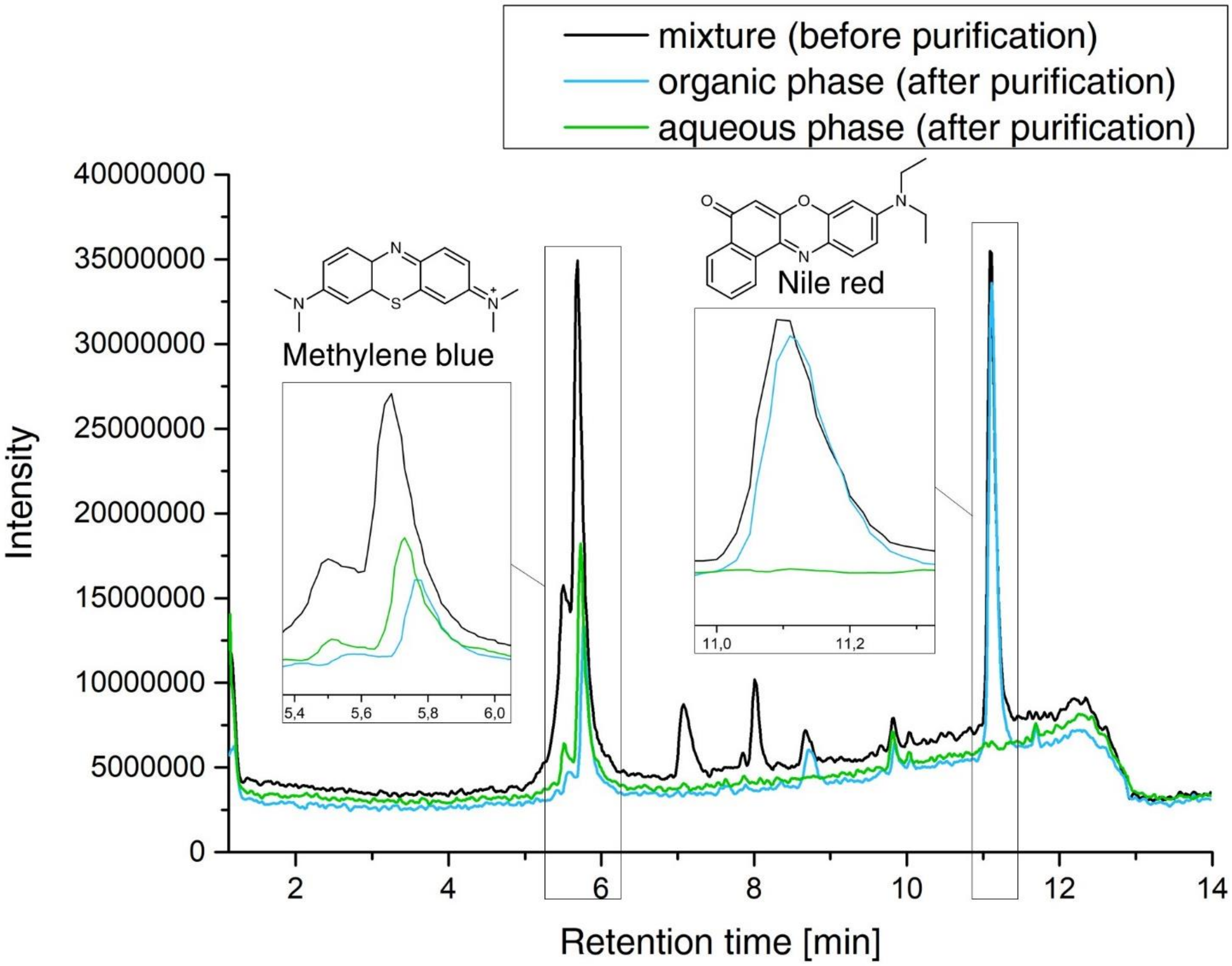

Supplementary Figure 9 | LC-MS characterization of the on-chip liquid-liquid extraction process. A mixture of a solution of Nile red and methylene blue was purified by an on-chip two-phase liquid extraction. The mixture (black line), the organic (red line) and the aqueous phase (blue line) was analyzed by LC-MS. Methylene blue showed a retention time of about 5.7 min, Nile red showed a retention time of 11.1 min. The intensity of methylene blue in the organic phase decreased after the purification step, while the intensity of Nile red remained the same. No peak for Nile red could be detected in the aqueous phase. Flow rate: 1 mL min^{-1}; detector: DAD (230 nm, 254 nm, 280 nm, 300 nm and 400 nm). Source data are provided as a Source Data file.

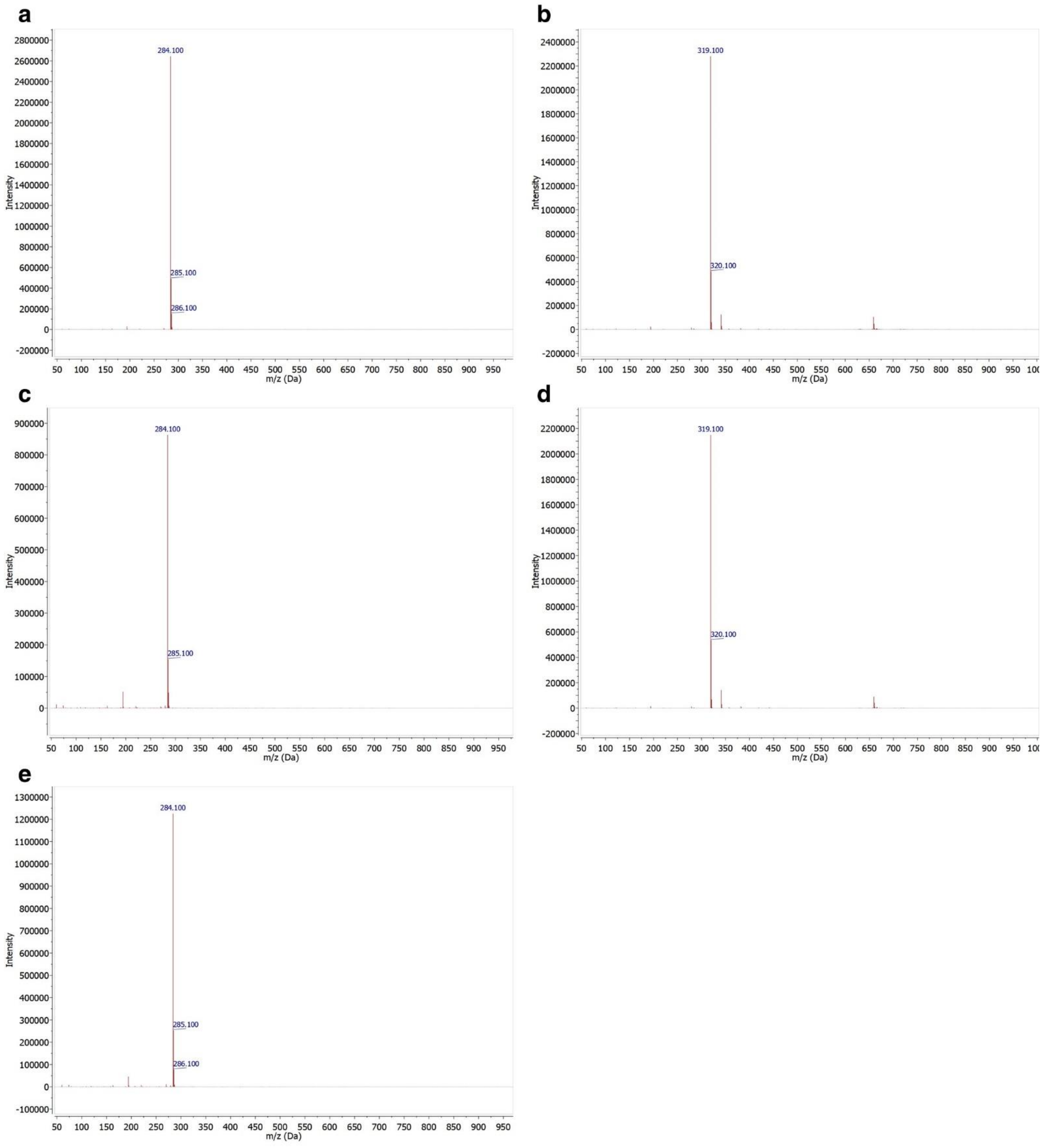

Supplementary Figure 10 | LC-MS spectra. (**a**) Mixture; retention time: 5.7 min – calculated mass (m/z): 284.12; found mass (m/z): 284.10. (**b**) Mixture; retention time: 11.1 min – calculated mass (m/z): 318.14; found mass (m/z): 319.10. (**c**) Organic phase; retention time: 5.8 min – calculated mass (m/z): 284.12; found mass (m/z): 284.10 (**d**) Organic phase; retention time: 11.1 min – calculated mass (m/z): 318.14; found mass (m/z): 319.10. (**e**) Aqueous phase; retention time: 5.7 min – calculated mass (m/z): 284.12; found mass (m/z): 284.10. Source data are provided as a Source Data file.

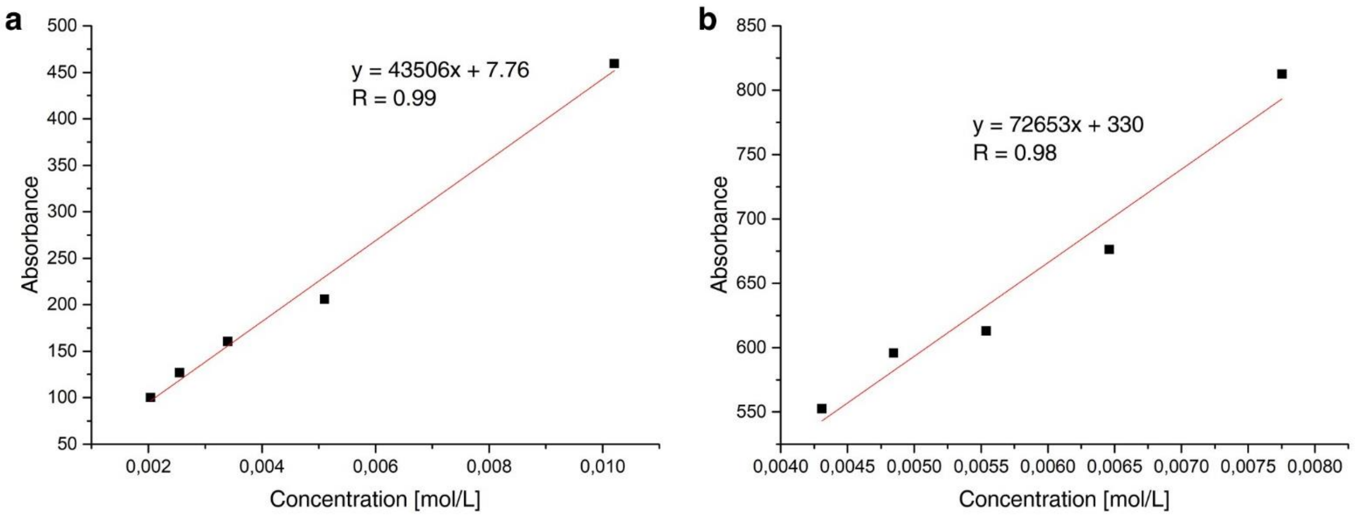

Supplementary Figure 11 | UV-Vis calibration curves. (**a**) Calibration curve of Nile red in acetonitrile. The extinction coefficient ε was estimated to be 43,506 $M^{-1}cm^{-1}$ at 541 nm. (**b**) Calibration curve of methylene blue in acetonitrile. The extinction coefficient ε was estimated to be 72,653 $M^{-1}cm^{-1}$ at 654 nm. Source data are provided as a Source Data file.

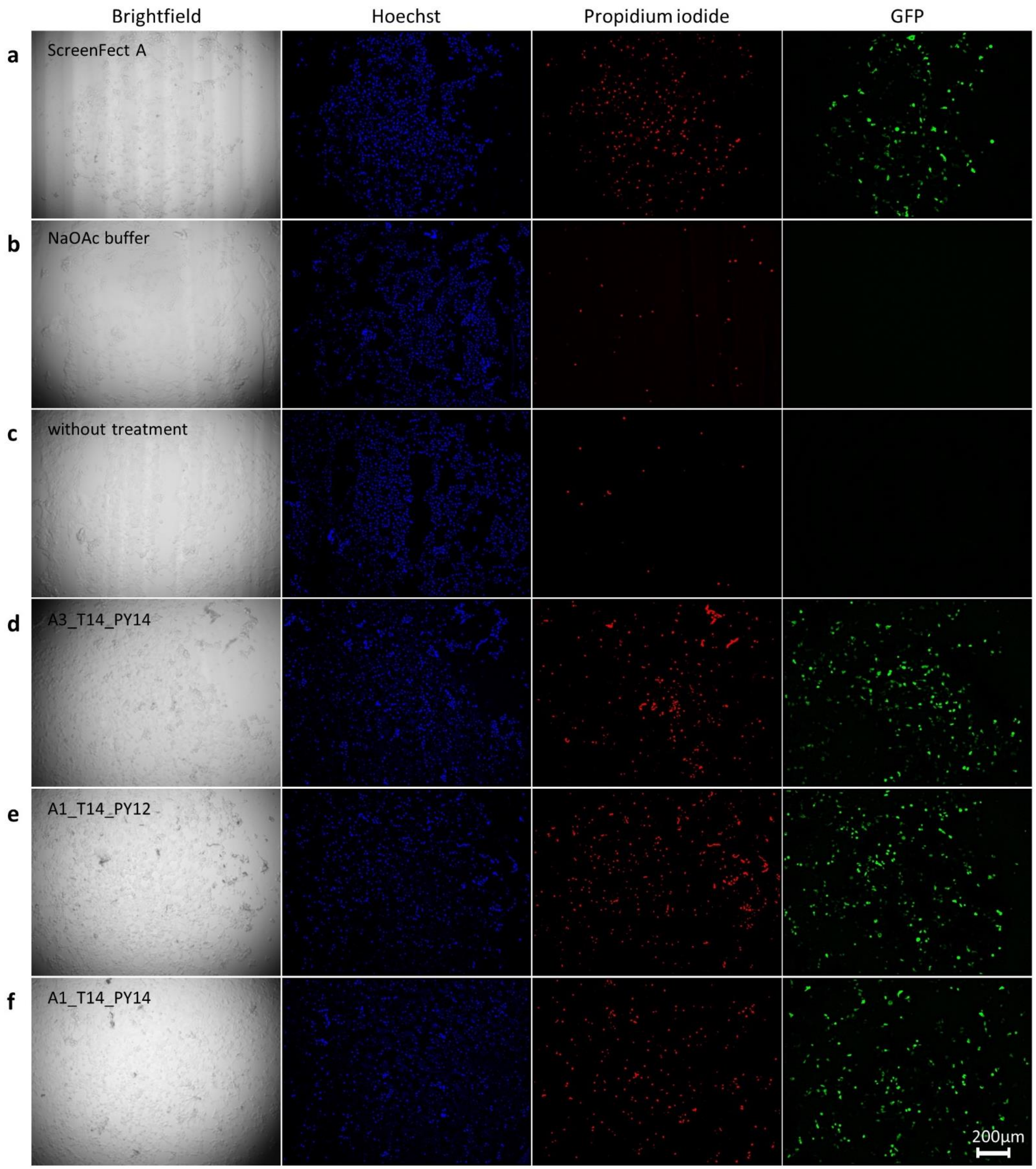

Supplementary Figure 12 | Exemplary microscope images of treated cells after 48h cultivation. From left to right: brightfield channel, Hoechst 33342 channel (λ_{ex} = 400 nm), propidium iodide channel (λ_{ex} = 595 nm), GFP channel (λ_{ex} = 505 nm); scale bar: 200 µm. (**a**) Cells treated with ScreenFect A as positive control. Transfection efficiency: 26%±1%; cell viability: 34%±1%. (**b**) Cells treated with aqueous sodium acetate buffer (50 mM, ph 5) as negative control. Transfection efficiency: 0%; cell viability: 97%±1%. (**c**) Cells without treatment as negative control. Transfection efficiency: 0%; cell viability: 98%±1%. (**d**) Cells treated with sample A3_T14_PY14. Transfection efficiency: 52%±4%; cell viability: 35%±11%. (**e**) Cells treated with sample A1_T14_PY12. Transfection efficiency: 50%±9%; cell viability: 36%±6%. (**f**) Cells treated with sample A1_T14_PY14. Transfection efficiency: 46%±7%; cell viability: 32%±9%. +/- values are standard deviations, n = 3 (number of replicates); N = 3 (repetitions including lipid synthesis). Source data are provided as a Source Data file.

Supplementary Table 1 | Synthesized lipidoid library and estimated yield of the reactions appreciated by UV-Vis absorbance measurements of the raw product. One equivalent of 2-thiopyridone corresponds to one equivalent of lipidoid. Each result based on triplicate control. Source data are provided as a Source Data file.

No.	ID	Product structure	Yield
1	A1_10_10	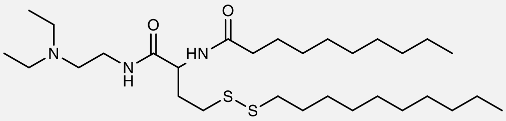	99±1
2	A1_12_12	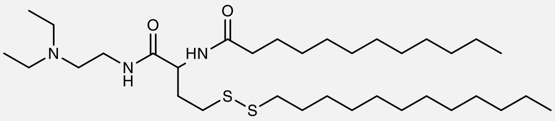	98±1
3	A1_14_14	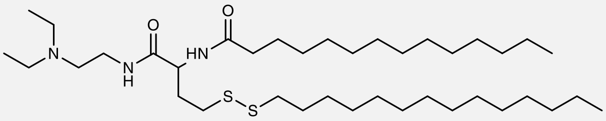	97±3
4	A1_14_10	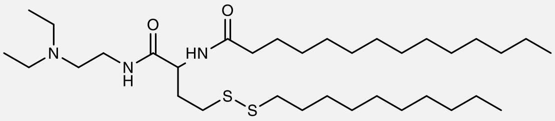	99±1
5	A1_14_12	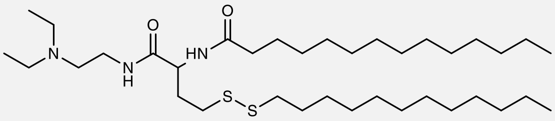	97±0
6	A2_10_10	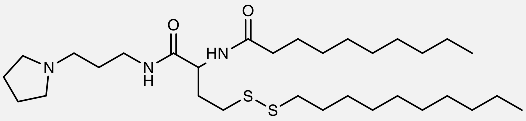	53±1
7	A2_12_12	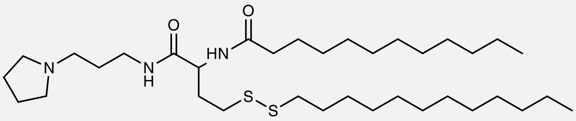	57±2
8	A2_14_14	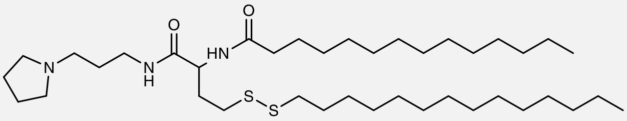	53±3
9	A2_14_10	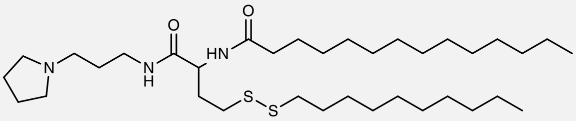	67±4
10	A2_14_12	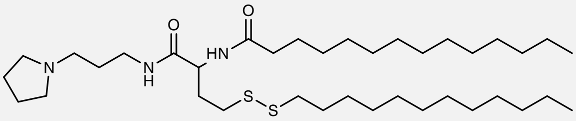	67±2
11	A3_10_10	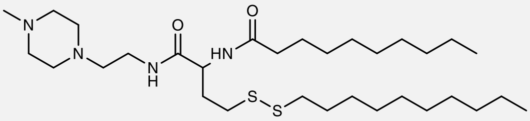	91±1
12	A3_12_12	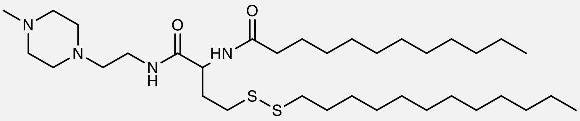	98±0
13	A3_14_14	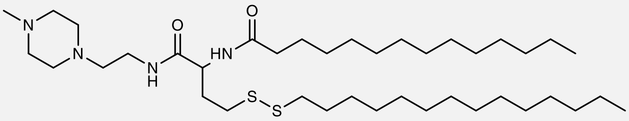	90±5
14	A3_14_10	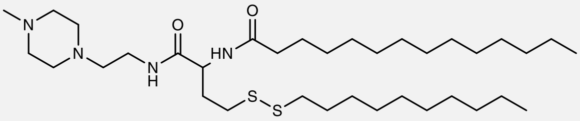	93±2

15	A3_14_12	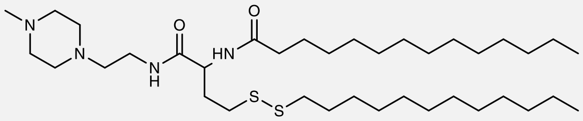	94±2
16	A4_10_10	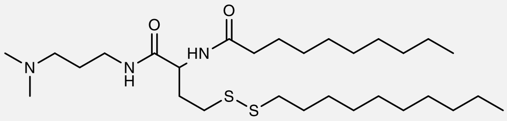	97±0
17	A4_12_12	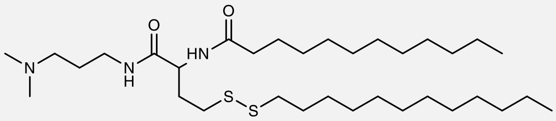	99±1
18	A4_14_14	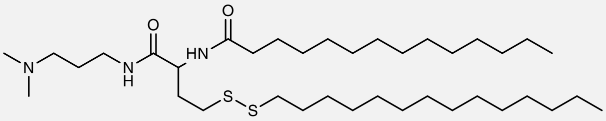	99±1
19	A4_14_10	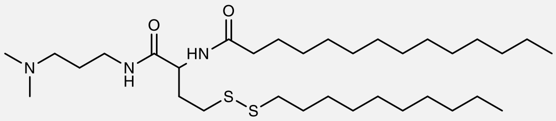	98±0
20	A4_14_12	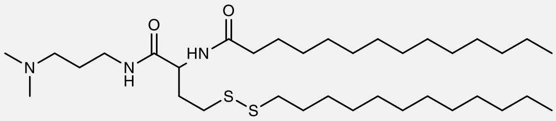	98±0
21	A5_10_10	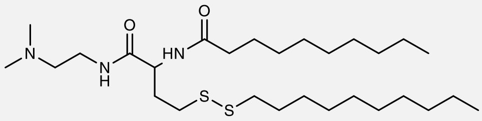	91±1
22	A5_12_12	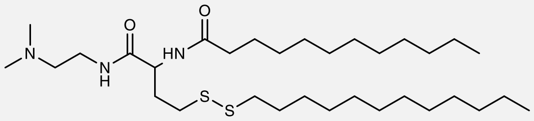	98±0
23	A5_14_14	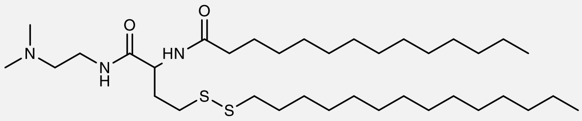	90±2
24	A5_14_10	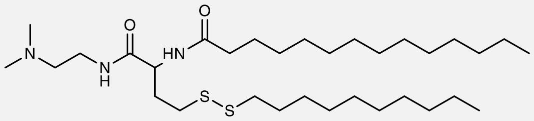	94±2
25	A5_14_12	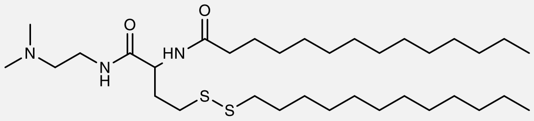	94±1

Supplementary Table 2 | Transfection efficiencies (TE) of test samples observed in three experiments (Exp.). Each result of a single experiment based on triplicate control. Source data are provided as a Source Data file.

No.	ID	TE(Exp. 1) [%]	TE(Exp. 2) [%]	TE(Exp. 3) [%]	Ø TE [%]	σ TE [%]
1	A1_10_10	0	0	0	0	0
2	A1_12_12	15	11	7	11	3
3	A1_14_10	43	17	12	24	14
4	A1_14_12	58	37	55	50	9
5	A1_14_14	51	36	51	46	7
6	A2_10_10	0	0	0	0	0
7	A2_12_12	0	0	0	0	0
8	A2_14_10	0	0	0	0	0
9	A2_14_12	0	0	0	0	0
10	A2_14_14	0	0	0	0	0
11	A3_10_10	0	0	0	0	0
12	A3_12_12	7	14	4	8	5
13	A3_14_10	36	44	50	43	6
14	A3_14_12	36	27	25	29	5
15	A3_14_14	53	56	46	52	4
16	A4_10_10	0	0	0	0	0
17	A4_12_12	9	0	1	3	4
18	A4_14_10	16	14	23	18	4
19	A4_14_12	8	5	12	8	3
20	A4_14_14	12	30	32	25	9
21	A5_10_10	0	0	0	0	0
22	A5_12_12	6	13	7	9	3
23	A5_14_10	36	35	35	36	0
24	A5_14_12	11	11	12	11	1
25	A5_14_14	30	39	29	33	5

Supplementary Table 3 | Calculated N/P values of selected lipoplexes. Source data are provided as a Source Data file.

Sample	N/P value
A1_T10_PY10	5:1
A3_T12_PY12	8:1
A4_T14_PY12	4:1
