# Supplementary material for: Marrying chemistry with biology by combining on-chip solution-based combinatorial synthesis and cellular screening
Source: Nat Commun. 2019 Jun 28;10:2879. doi: 10.1038/s41467-019-10685-0 (PMC6599004; doi:10.1038/s41467-019-10685-0)
Supplement: Supplementary file 4 — Reporting Summary [file 41467_2019_10685_MOESM4_ESM.pdf]

nature research

Corresponding author(s): Dr. Pavel A. Levkin

Last updated by author(s): May 13, 2019

Reporting Summary

Nature Research wishes to improve the reproducibility of the work that we publish. This form provides structure for consistency and transparency in reporting. For further information on Nature Research policies, see Authors & Referees and the Editorial Policy Checklist.

Statistics

For all statistical analyses, confirm that the following items are present in the figure legend, table legend, main text, or Methods section.

n/a	Confirmed	
☐	☒	The exact sample size (*n*) for each experimental group/condition, given as a discrete number and unit of measurement
☐	☒	A statement on whether measurements were taken from distinct samples or whether the same sample was measured repeatedly
☒	☐	The statistical test(s) used AND whether they are one- or two-sided<br>*Only common tests should be described solely by name; describe more complex techniques in the Methods section.*
☒	☐	A description of all covariates tested
☒	☐	A description of any assumptions or corrections, such as tests of normality and adjustment for multiple comparisons
☐	☒	A full description of the statistical parameters including central tendency (e.g. means) or other basic estimates (e.g. regression coefficient) AND variation (e.g. standard deviation) or associated estimates of uncertainty (e.g. confidence intervals)
☒	☐	For null hypothesis testing, the test statistic (e.g. *F*, *t*, *r*) with confidence intervals, effect sizes, degrees of freedom and *P* value noted<br>*Give P values as exact values whenever suitable.*
☒	☐	For Bayesian analysis, information on the choice of priors and Markov chain Monte Carlo settings
☒	☐	For hierarchical and complex designs, identification of the appropriate level for tests and full reporting of outcomes
☒	☐	Estimates of effect sizes (e.g. Cohen's *d*, Pearson's *r*), indicating how they were calculated

Our web collection on statistics for biologists contains articles on many of the points above.

Software and code

Policy information about availability of computer code

Data collection	Water Contact Angle: Krüss Advance 1.6.2.0; Krüss GmbH<br>MS: 4000 Series Explorer Software V 3.5<br>NMR: TopSpin 3.5 pl 6 (build 1784); Bruker BioSpin<br>UV-Vis: UV WinLab 6.0.4; PerkinElmer, Inc.<br>DLS/Zeta: Zetasizer Software 7.11; Malvern Instruments Ltd.<br>Microscopy: BZ-II Viewer 1.5.0.0; Keyence Corporation
Data analysis	Water Contact Angle: Krüss Advance 1.6.2.0; Krüss GmbH<br>MS: Data Explorer Software V 4.9 (build 115)<br>NMR: ACD/Spectrus Processor 2015 (build 74482); Advanced Chemistry Development, Inc. / MestReNova v9.0.1-13254; Mestrelab Research S.L.<br>UV-Vis: Excel 2016 Build 9126.2259; Microsoft / OriginPro 9.1.0G; OriginLab Corporation<br>Microscopy: BZ-II Analyzer 2.1; Keyence Corporation / ImageJ 1.51s; Wayne Rasband National Institutes of Health / Excel 2016 Build 9126.2259; Microsoft / OriginPro 9.1.0G; OriginLab Corporation<br>LC-MS: OpenChrom Comunity Edition Version 1.3.0.201808081331 / OriginPro 9.1.0G; OriginLab Corporation

For manuscripts utilizing custom algorithms or software that are central to the research but not yet described in published literature, software must be made available to editors/reviewers. We strongly encourage code deposition in a community repository (e.g. GitHub). See the Nature Research guidelines for submitting code & software for further information.

Data

Policy information about availability of data

All manuscripts must include a data availability statement. This statement should provide the following information, where applicable:

- Accession codes, unique identifiers, or web links for publicly available datasets
- A list of figures that have associated raw data
- A description of any restrictions on data availability

The data that support the findings of this study are available from the corresponding author upon reasonable request.

Field-specific reporting

Please select the one below that is the best fit for your research. If you are not sure, read the appropriate sections before making your selection.

☒ Life sciences ☐ Behavioural & social sciences ☐ Ecological, evolutionary & environmental sciences

For a reference copy of the document with all sections, see nature.com/documents/nr-reporting-summary-flat.pdf

Life sciences study design

All studies must disclose on these points even when the disclosure is negative.

Sample size	Because our study presents a novel method for combining synthesis of bioactive compounds with their biological readout, no sample size calculation were performed to predetermine the sample size. Stock solutions of each educt for the synthesis of lipidoid derivates were diluted and mixed at the same concentration for each replication. The repeated experiment times were sufficient to demonstrate the reproducibility and validity of our new method.
Data exclusions	No data were excluded from the analysis.
Replication	All attempts at replication were successful. The repeating numbers for each experiment are stated in the method part where it is applicable.
Randomization	The experiments were not randomized.
Blinding	Blinding was not used for the experiments.

Reporting for specific materials, systems and methods

We require information from authors about some types of materials, experimental systems and methods used in many studies. Here, indicate whether each material, system or method listed is relevant to your study. If you are not sure if a list item applies to your research, read the appropriate section before selecting a response.

Materials & experimental systems

n/a	Involved in the study	
☒	☐	Antibodies
☐	☒	Eukaryotic cell lines
☒	☐	Palaeontology
☒	☐	Animals and other organisms
☒	☐	Human research participants
☒	☐	Clinical data

Methods

n/a	Involved in the study	
☒	☐	ChIP-seq
☒	☐	Flow cytometry
☒	☐	MRI-based neuroimaging

Eukaryotic cell lines

Policy information about cell lines

Cell line source(s)	293T (ATCC® CRL3216™)
Authentication	Authentication of the cell line by supplier.
Mycoplasma contamination	All cell lines tested negative for mycoplasma contamination.
Commonly misidentified lines (See ICLAC register)	No misidentified cell lines were used.
